# A novel marker in the ovarian preservation approach to endometrial cancer: systemic immune inflammatory index

**DOI:** 10.61622/rbgo/2025rbgo59

**Published:** 2025-09-08

**Authors:** Büşra Şahin, Tansu Bahar Gürbüz, Ayşe Sinem Duru Çöteli, Emel Ebru Begen, Arife AKAY, Nurettin Boran, Yaprak Üstün

**Affiliations:** 1 Ankara Etlik Zübeyde Hanım Women's Health Training and Research Hospital Department of Obstetrics and Gynecology Ankara Turkey Department of Obstetrics and Gynecology, Ankara Etlik Zübeyde Hanım Women's Health Training and Research Hospital, Ankara, Turkey.; 2 Duzce Ataturk State Hospital Department of Obstetrics and Gynecology Duzce Turkey Department of Obstetrics and Gynecology, Duzce Ataturk State Hospital, Duzce, Turkey; 3 Ankara Health Science University Etlik Zubeyde Hanim Women's Health and Research Hospital Department of Gynecological Oncology Ankara Turkey Department of Gynecological Oncology, Ankara Health Science University Etlik Zubeyde Hanim Women's Health and Research Hospital, Ankara, Turkey.

**Keywords:** Endometrial neoplasms, Ovarian neoplasms, Leukocytes, Neutrophils, Blood platelets, Organ preservation, Systemic immune inflammatory index

## Abstract

**Objective:**

This study aims to to evaluate the frequency of ovarian involvement in endometrial cancer patients aged 50 years and younger, identify associated clinicopathological factors, and uniquely assess the role of the Systemic Immune-Inflammatory Index (SII) in predicting ovarian involvement.

**Methods:**

Patients aged 50 years and younger diagnosed with endometrial cancer between 1992 and 2022 were retrospectively analyzed. Two groups were formed based on adnexal involvement: those with (ovarian metastasis or synchronous ovarian cancer) and without adnexal involvement. Clinicopathological predictors of adnexal involvement were evaluated. Preoperative complete blood count values (platelet, leukocyte, lymphocyte, and neutrophil counts) were used to calculate inflammatory indices: PLR (platelet-to-lymphocyte ratio), NLR (neutrophil-to-lymphocyte ratio), and SII (neutrophil × platelet / lymphocyte). A two-group analysis was performed based on the cut-off values of statistically significant parameters. Univariate and multivariate logistic regression analyses were conducted.

**Results:**

Among 205 patients, histopathological ovarian metastasis was identified in 5.9% (n=12), and synchronous ovarian tumors in 2.4% (n=5). Significant differences were observed in neutrophil counts, NLR, and SII values between the groups (p<0.05). ROC analysis showed the optimal SII cut-off value as 992.58, with 70% sensitivity and 76% specificity (AUC=0.726). Ovarian involvement was significantly more frequent in patients with SII ≥ 992 (p<0.05). Univariate analysis revealed that myometrial invasion, LVSI, cervical stromal invasion, lymph node metastasis, omental involvement, grade of tumor, NLR and SII were significantly associated with ovarian involvement (p<0.05). Multivariate analysis identified histological grade, myometrial invasion, pelvic lymph node metastasis and SII as independent risk factors (p<0.05).

**Conclusion:**

Ovarian involvement is uncommon in patients under 50 years of age with low-grade tumors, absence of myometrial invasion, negative pelvic lymph nodes, and preoperative SII < 992.58. Ovarian-sparing surgery may be a safe option in selected cases, and SII could serve as a valuable index in guiding ovarian preservation decisions.

## Introduction

Endometrial cancer (EC) is the most common gynecologic malignancy worldwide with an age-standardized prevalence of 8.4% following cervical cancer. In Turkey, it is the most common gynecologic malignancy with a prevalence of 6.1% in all age groups and 3.8% in women aged 25-49 years.^(1,2)^

Five percent of the patients are diagnosed before age 40, and the incidence of early age EC is increasing.^(3–5)^ Incidence of adnexal metastases in EC ranges from 2% to 8.1% and are usually characterized by ovarian pathologies that can be detected during surgery.^(6–9)^ According to the literature, microscopic metastasis is less than 1% in patients with adnexal involvement. Synchronous ovarian cancer is seen in 2% of the patients, and these patients usually have abnormal ovarian morphology.^(10–13)^

Surgical staging is done in EC, and hysterectomy with bilateral salpingo-oophorectomy (BSO) is the standard treatment due to the risk of microinvasive ovarian involvement. In the early period, due to estrogen deficiency, vasomotor symptoms, sexual dysfunction, sleep disorders, and mood changes may be observed in patients undergoing BSO. In the long term, patients are at an increased risk for osteoporosis and cardiovascular diseases.^(14–19)^

These problems, which are secondary to oophorectomy, brought the ovarian preservation approach into question for the young patient group. When studies in the literature are examined, there is no clear consensus on the subject, especially due to the risk of micrometastasis to the ovary. However, one of the most recent reviews concluded that an ovarian-sparing approach can be applied to patients with FIGO stage 1A, grade 1-2, endometrioid type, and those younger than 40 years.^(20)^ Disease confined to the pelvis, disease microscopically limited to the uterus and ovary, and low histologic grade are good prognostic factors for synchronous ovarian cancer. In addition, deep myometrial invasion on preoperative imaging, high ca125 levels and positive adnexal involvement on MRI/ 3urgical exploration are among the independent predictive factors for coesting adnexal malignancy.^(9,10)^

SII is considered a good index of local immune response and systemic inflammation based on peripheral lymphocyte, neutrophil and platelet counts. In recent years, there has been increasing interest in the tumor microenvironment. Inflammatory changes in the tumor microenvironment are known to have an impact on cancer cell proliferation, metastasis, angiogenesis, and immune escape.^(21,22)^

SII has an important role in survival and prognosis in gynecologic malignancies. In a recent study, high SII was presented as an independent risk factor for postmenopausal advanced EC.^(23)^ On the other hand, ovarian involvement is known to be important in EC prognosis. However, there is no study in the literature investigating the relationship between ovarian involvement and SII. Therefore, knowing the adnexal participation incidence and synchronous ovarian cancer in endometrial cancer patients and defining the parameters to predict the adnexal participation will guide the decision for an ovarian preservation approach.

This study aims to determine the frequency of ovarian involvement in patients aged 50 years and younger, investigate the associated factors, and, unlike other studies in the literature, investigate the role of the systemic immune-inflammatory index (SII) in ovarian involvement.

## Methods

All the patients diagnosed with endometrial cancer who received primary surgical treatment at the Gynecological Oncology Clinic of the Health Sciences University Etlik Zübeyde Hanım Gynecology and Obstetrics Training and Research Hospital between 1992 and 2022 were retrospectively scanned using the hospital's information system. Patients aged 50 and under were included in the study, and their demographic characteristics and pathology reports were reviewed. Patients were examined in two groups: patients with adnexal involvement (ovarian metastasis and synchronous ovarian cancer) and patients without adnexal involvement. Information on histological type (endometrioid, non-endometrioid), histological grade (grade 1, grade 2-3), FIGO (International Federation of Gynecology and Obstetrics, 2009) stage (stage 1-2, stage 3-4), myometrial invasion (no MI, <50% MI, >50% MI), lymphovascular invasion (LVSI), endocervical glandular involvement, cervical stromal involvement, lymph node metastasis, omental involvement, and cytology were recorded. Clinicopathological parameters that can be used to predict the adnexal participation were analyzed in both groups.

Complete blood count parameters (platelet, leukocyte, lymphocyte, neutrophil) obtained from the preoperative patients were analyzed using the Mindray BC-6000 device. To evaluate inflammatory indices, PLR (platelet/lymphocyte), NLR (neutrophil/lymphocyte), and SII (neutrophil x platelet/lymphocyte) were calculated, and the values were recorded in the patient's follow-up form. A two-group analysis was performed between the groups by determining the cut-off value for the statistically significant parameters. For the laboratory parameters, cut-off values were determined for the variables that were significant using a ROC analysis. A two-group analysis was also performed. Independent predictive factors were investigated by performing a multivariate logistic regression analysis for parameters that were significant in the univariate analysis between both groups.

Analyzes were done with the SPSS 21.0 program and were studied at a confidence level of 95%. The kurtosis and skewness values obtained from the measurements between +3 and -3 were sufficient for the normal distribution. Numbers (n) and percentages (%) were calculated for categorical variables and mean standard deviation (SD) was calculated for numerical variables. Statistical significance was taken as p<0.05. Parametric variables were analyzed with a T-test or Mann Whitney U Test according to the normal distribution for the two independent groups. The relationship between categorical variables was analyzed with the Chi-square test. Moreover, the results of multivariate analysis and ORs of logistic regression analysis structures between groups for categorical variables were also presented. Predictive factors were analysed with a multivariate logistic regression analysis. The receiver operating characteristic (ROC) curve was used to show the sensitivity and specificity of SII and NLR. The detection value of β-hCG increases to 1 when the area under the curve (AUC) value is greater than 0.5. Also, the appropriate equation was created using regression analysis for SII and NLR.

For this retrospective study, permission numbered 2024/11 was received from the Local Ethics Committee of the Health Sciences University Etlik Zübeyde Hanım Gynecology and Pediatrics Training and Research Hospital 30.10.2024.

## Results

A total of 205 patients were included in the study, and histopathological ovarian metastasis was detected in 5.9% (n=12), and synchronous ovarian tumor was detected in 2.4% (n=5). The median age of the patients was 44.6±4.7 years. No statistically significant difference was found in ovarian involvement between those under and over 45 (p: 0.917). Clinicopathological features of the patients with and without ovarian involvement are presented in table 1. In the univariate analysis, non-endometrioid histological type, increased grade, advanced stage disease, myometrial invasion depth, LVSI, cervical stromal invasion, lymph node metastasis, and omental involvement indicate an increased risk for ovarian involvement (p<0.05). Preoperative hematologic parameters are shown in Table 2. A statistically significant difference was found in neutrophil count (p = 0.015), NLR (p = 0.020), and SII (p = 0.023) between the two groups. Mean SII was significantly higher in the group with adnexal malignancy (1201.62 ± 631.95 vs. 808.54 ± 586.96).

**Table 1:** Comparison of clinicopathologic risk factors between patients with and without coexisting adnexal malignancies

Variables		No coexisting malignancy Group 1 n=188	Coexisting malignancy Group 2 n=17	p-value
		n(%)	n(%)	
Age				
	≤ 45	86(91.5)	8(8.5)	0.917
	46-50	102(91.9)	9(8.1)	
Histologic subtype				
	Endometrioid	163(94.2)	10(5.8)	0.002
	Non-endometrioid	25(78.1)	7(21.9)	
Histologic grade				
	Grade 1	150(96.2)	6(3.8)	<0.001
	Grade 2-3	38(77.6)	11(22.4)	
Stage				
	1-2	172(98.3)	3(1.7)	<0.001
	3-4	16(53.3)	14(46.7)	
Myometrial invasion				
	No	67(97.1)	2(2.9)	<0.001
	Yes	121(88.9)	15(11.1)	
LVSI				
	No	167(94.9)	9(5.1)	<0.001
	Yes	21(72.4)	8(27.6)	
Endocervical glandular involvement				
	No	180(90.1)	16(8.2)	0.754
	Yes	8(88.9)	1(11.1)	
Cervical stromal invasion				
	No	177(94.1)	11(5.6)	<0.001
	Yes	11(64.7)	6(35.3)	
Metastases to pelvic lymph nodes				
	No	175(95.6)	8(4.4)	<0.001
	Yes	13(59.1)	9(40.9)	
Metastases to para-aortic lymph nodes				
	No	181(93.8)	12(6.2)	<0.001
	Yes	7(58.1)	5(41.7)	
Omental involvement				
	No	186(93)	14(7)	<0.001
	Yes	2(40)	3(60)	

LVSI - Lymphovascular Space Invasion; Chi-Square; Fisher's Exact Test

**Table 2:** Comparison of inflammatory markers between patients with and without coexisting adnexal malignancies

	No coexisting malignancy Group 1 n:121	Coexisting malignancy Group 2 n:10	p-value
Platelets (x103/mm3)	309.52±102.92	330.18±101.66	0.436**
Leukocytes (x10^3^/mm^3^)	7.65± 2.49	9.04± 2.34	0.051*
Lymphocytes (x10^3^/mm^3^)	2.06± 0.68	1.99±1.06	0.369*
Neutrophils (x10^3^/mm^3^)	4.85± 2.08	6.19±1.59	0.015*
NLR (%)	2.55± 1.26	3.59±1.34	0.020**
PLR (%)	163.55± 70.38	203.09± 115.53	0.054**
SII	808.54± 586.96	1201.62± 631.95	0.023*

NRL - Neutrophil Lymphocyte Ratio; PLR - Platelet Lymphocyte Ratio; SII - Systemic Inflammatory Index

* t-test;

** Mann Whitney test

ROC analysis results are summarized in table 3. The area under the curve (AUC) was 0.751 for NEU, 0.738 for NLR, and 0.726 for SII, indicating good diagnostic performance. The optimal cut-off values were: NEU ≥ 5.02 (80% sensitivity, 64.5% specificity), NLR ≥ 2.63 (80% sensitivity, 64.5% specificity), and SII ≥ 992.58 (70% sensitivity, 76% specificity). When patients were grouped according to the SII cut-off value of 992.58, adnexal malignancy was found to be significantly more frequent in those with higher SII values (p < 0.05).

**Table 3:** Receiver Operating Characteristic (ROC) curve analysis of NEU, NLR, and SII for predicting coexisting adnexal malignancies

Test result variable(s)	Area	Sth. error	Asymptotic Sig.	Asymptotic 95% Confidence Interval	
				Lower bound	Upper bound
NEU	0.751	0.067	0.008	0.620	0.883
NLR	0.738	0.086	0.013	0.569	0.907
SII	0.726	0.076	0.018	0.576	0.875

The histopathological features and intraoperative findings of patients with ovarian involvement are evaluated in table 4. While normal ovarian morphology was observed in 6 patients, extrauterine spread was present in 83% of the patients with normal ovarian morphology. Only in one patient older than 45 years of age, despite normal ovarian morphology, no intraoperative spread outside the uterus was detected. There were patients younger than 45 years of age, having advanced disease in the presence of micrometastatic ovaries, indicating extrauterine spread. Intraoperative ovarian morphology was abnormal in all the cases with synchronous ovarian cancer (Table 4). Univariate analysis revealed that myometrial invasion, LVSI, cervical stromal invasion, lymph node metastasis, omental involvement, grade of tumor, NLR and SII were significantly associated with ovarian involvement (p<0.05) (Table 5).

**Table 4:** Clinicopathological characteristics of patients with adnexal involvement

Patient	Age	Site	HS	Grade	FIGO Stage	Ovarian/tubal morphology	LVSI	Endocervical glandular ınvolvement	Cervical stromal invasion	Major intraoperative findings
M	45	LO	E	3	3	Normal	-	+	+	Cervical mass, Palpable LN
M	33	LO	S	1	3	Normal	-	-	-	Left cornual surface,5*5 cm multilobular tumural vegation, Palpable LN, Acid
M	47	BO	S	3	4	Normal	+	+	+	Peritoneal and omental seeding, Palpable LN
M	48	RO	E	3	4	Normal	-	-	+	Peritoneal seeding (rectosigmoid, douglas), Palpable LN
M	42	RO	E	3	3	Abnormal	-	-	+	RO; tumoral infiltration, Palpable LN
M	46	BO	E	3	3	Abnormal	+	-	-	LO, 7*9 cm cystic and solid; RO, 4*5 cm cystic and solid, Palpable LN
M	42	RS	E	3	3	Abnormal	+	-	-	Abnormal salpinx
M	47	LO	E	2	3	Abnormal	+	-	-	LO; 3*3cm cystic and solid
S	50	RO	M	2	3	Abnormal	+	-	-	RO; 5*6cm cystic and solid
S	43	BO	S	1	1	Abnormal	+	-	-	Bilateral TOA
M	47	RO	E	1	3	Normal	-	-	-	-
M	49	BO	E	2	3	Abnormal	-	-	+	RO; 5*6 cm cystic and solid, LO; 10*15 cm cystic and solid
M	48	LO	U	3	3	Normal	+	-	+	Palpable LN
S	43	BO	E	1	1	Abnormal	-	-	-	RO; solid and cyst, tumoral infiltration
M	44	BO	E	1	4	Abnormal	+	-	-	Bilateral large ovary, LH, Palpable LN
S	46	BO	S	3	4	Abnomal	-	-	-	LO; 20*15 cm cystic and solid, RO; 5*6 cm cystic and solid, serosal tumoral infiltration (rectum)
S	44	RO	E	1	1	Abnormal	-	-	-	Large ovary, tumoral infiltration

En - endometrioid; S - carcinosarcoma; U - undifferentiated; M - mixed; RO - right ovary; LO - left ovary; RS - Right hydrosalpinx; TOA - Tubal ovarian abscess; LN - lymph node; LH - Left hydrosaliınx; LVSI - lymphovascular space invasion

**Table 5:** Univariate and multivariate logistic regression analyses of clinicopathologic factors associated with coexisting adnexal malignancies

Factor		Univariate logistic regression	p-value*	Multivariate logistic regression	p-value*
		Odds ratio (95% CI)		Odds ratio (95% CI)	
Stage					
	Stage 1-2	1 (reference)			
	Stage 3-4	39.2 (10.36-148.26)	<0.001		
Myometrial invasion					
	No	1 (reference)			
	Yes	13.98 (4.66-41.88)	<0.001	12.4 (1.49-45.92)	0.046
LVSI				
	No	1 (reference)			
	Yes	7.06 (2.46-20.3)	<0.001	-	-
Cervical stromal invasion					
	No	1 (reference)			
	Yes	8.77 (2.73-28.17)	<0.001	-	-
Metastases to pelvic lymph nodes					
	No	1 (reference)			
	Yes	14.19 (4.69-42.94)	<0.001	30.54 (1.96-475.72)	0.015
Metastases to para-aortic lymph nodes					
	No	1 (reference)			
	Yes	9.16 (2.52-33.27)	0.001	-	-
Omental involvement					
	No	1 (reference)			
	Yes	17.25 (2.65-112)	0.003	-	-
Histologic grade					
	Grade 1	1 (reference)			
	Grade 2-3	8.94 (3.05-26.19)	<0.001	19.7 (1.17-350.6)	0.042
NLR					
	< 2.63	1 (reference)			
	≥ 2. 63	7.25 (1.47-35.7)	0.015	-	-
SII					
	< 992.58	1 (reference)			
	≥992.58	7.4 (1.79-30.48)	0.006	1.45 (1.01-12.34)	0.034

* SII - Systemic immune inflammation index;

LVSI - Lymphovascular space involvement; NLR - Neutrophil to lymphocyte ratio. Logistic regression analysis

In the multivariate logistic regression analysis (Table 5), myometrial invasion (OR: 12.4; 95% CI: 1.49–45.92; p=0.046), pelvic lymph node metastasis (OR: 30.54; 95%CI: 1.96–475.72; p=0.015), tumor grade (OR: 19.7; 95%CI: 1.17–350.6; p=0.042), and high SII (≥992.58) (OR: 1.45; 95%CI: 1.01–12.34; p=0.034) remained as independent predictors of adnexal involvement.

## Discussion

One of the most critical problems in the management of the premenopausal patient group of EC patients is the early and late consequences of surgical menopause secondary to bilateral salpingo-oophorectomy included in the standard treatment. The ovarian preservation approach for these patients is one of the controversial issues due to ovarian micrometastasis. Therefore, it is crucial to investigate the incidence of ovarian involvement in EC patients, to identify the risk factors for ovarian participation, and to determine the parameters to be used in selecting suitable patients for ovarian-sparing surgery.

The most recent study on the subject reported that ovarian metastasis occurred at incidences ranging from 2% to 8.1% for the patients who underwent oophorectomy due to endometrial cancer.^(20)^ Moreover, studies that only included patients diagnosed with EC under the age of 50 show that ovarian metastases were detected at incidences ranging from 1-8%, and synchronous ovarian cancer is detected at 3-5%.^(3,8)^

In this study, it is found that ovarian metastasis was seen in 5.9% of the patients and synchronous ovarian involvement was seen in 2.4% of the patients, and these findings are consistent with the literature. Six of the patients had micrometastatic ovarian involvement, and extrauterine metastasis was observed in 83% of the patients in whom micrometastatic ovaries were detected. Micrometastatic ovaries were detected in only one patient over 45 years of age, although the ovaries were normal, and there was no extrauterine spread. The micrometastatic ovarian involvement rate in this study is above the incidence in the literature, and according to the literature, this rate is below 1% in other conducted studies.^(3–5)^

In the ovarian preservation approach, it is essential to determine the criteria to be used in appropriate patient selection and to determine the risk factors for ovarian involvement due to the proximity of the uterus and ovary, the risk of micrometastasis to the ovary, and primary ovarian cancer in the following years. In a study evaluating the advantages and disadvantages of the ovarian preservation approach, a two-stage evaluation was recommended in patient selection; it was revealed that the ovarian preservation approach could be preferred in patients with no family history of breast or ovarian cancer, no risk for Lynch syndrome, and no intraoperative findings (extrauterine metastasis, upgrade or high-grade, deep myometrial invasion, cervical invasion, large tumor, etc.).^(3)^ In 2018, the National Comprehensive Cancer Network (NCCN)^(24)^ reported that ovarian preservation is possible in patients with no family history of ovarian or breast cancer, no Lynch syndrome, and macroscopically normal ovarian appearance. Baiocchi et al.^(25)^ demonstrated that the ovarian preservation approach can be considered in patients with low-grade (grade 1-2) disease, absence of LVSI, myometrial invasion of <50%, and under 45 years of age. In a most recent review in 2023, Bizzarri et al.^(20)^ discussed the ovarian preservation approach in gynecological cancers. They concluded that the ovarian preservation approach could be applied to patients with FIGO stage 1A, grade 1-2, endometrioid type, and younger than 40.^(20)^ In our study, we found that advanced increased grade, depth of myometrial invasion and metastatic pelvic lymph node involvement were independent prognostic factors. This study found that non-endometrioid histological type, increased grade, advanced stage of the disease, myometrial invasion depth, LVSI, cervical stromal invasion, lymph node metastasis, and omental involvement were significant in predicting ovarian involvement. Among these variables, it is found that the grade, myometrial invasion and metastatic pelvic lymph node involvement were independent prognostic factors. When patients with ovarian involvement were examined, 83% of the patients with micrometastatic ovarian involvement were over 45 years of age. For this reason, patient selection for ovarian-sparing surgery should be done carefully in the patient group over 45 years of age. In their meta-analysis evaluating the risk factors for ovarian metastasis, Liang et al.^(26)^ reported that data on ovarian protection in the postmenopausal patient group were lacking and that being over 45 remained a significant risk factor for ovarian recurrence. One of the critical questions in ovarian conservation approach is survival. The literature, reports no statistically significant survival difference between patients who have undergone salpingo-oophorectomy and those who have not.^(27–31)^ Additionally, some studies suggest that patients who undergo ovarian-sparing surgery have better disease-free survival, but similar overall survival.^(32)^

SII is a new index that reflects local immune response and systemic inflammation based on peripheral lymphocyte, neutrophil, and platelet counts. Especially recent studies show that it is effective in tumor prognosis and survival. Inflammatory changes in the tumor microenvironment contribute to cancer cell proliferation, metastasis, angiogenesis, and immune escape. While lymphocytes are valuable in generating tumor immunity, neutrophil cells are the effector cells of the acute inflammatory response. During a systemic infection in the body, platelet counts increase and aggravate tumor growth. A decrease in the number of lymphocytes results in decreased tumor immunity. Factors that stimulate granulopoiesis and thrombopoiesis in tumor cells are associated with elevated SII secondary to systemic inflammation in EC patients.^(21,22,33,34)^ SII plays an important role in survival and prognosis in gynecologic malignancies. In a recent study, high SII was presented as an independent risk factor for postmenopausal advanced EC.^(23)^ It is known that ovarian involvement is important in EC prognosis. However, there is no study in the literature examining the relationship between ovarian involvement and SII.

This study is the first to investigate the relationship between ovarian involvement in preoperative SII and EC. When the studies in the literature investigating the relationship between endometrial cancer and systemic immune-inflammatory index were analyzed, it was revealed that postoperative SII plays a role as an independent prognostic factor in survival and was also an independent risk factor in lymph node metastasis. It was also identified as an essential inflammatory marker in predicting myometrial invasion and high-grade cancer in the young patient population.^(23,27)^ In our study, which revealed a close relationship between ovarian involvement and SII in EC patients, we presented high SII as an independent prognostic factor for ovarian involvement when patients were grouped according to a cut-off value of 992. According to the results of this study, SII should be considered an index that can be used in patient selection in the ovarian preservation approach.

The strengths of this study include being the first to discuss the role of SII in ovarian involvement in endometrial cancer, including patients aged 50 and under as a sample, and supporting the literature in selecting patients for ovarian-sparing surgery. The study's limitations include its retrospective design, few patients and not using the FIGO 2023 staging system.

## Conclusion

Due to the adverse effects of early menopause on women's health, including vasomotor symptoms, impaired bone health, dyslipidemia, and increased cardiovascular risk, ovarian preservation has gained attention in the surgical management of endometrial cancer. Although bilateral salpingo-oophorectomy (BSO) is a standard component of treatment—primarily due to the risk of ovarian micrometastasis, proximity of the ovaries to the uterus, and the persistence of endogenous hormone production—its long-term consequences must be carefully weighed. According to our findings, ovarian-sparing surgery may be considered a viable option in patients under 50 years of age, with early-stage, low-grade tumors, no myometrial invasion, and no pelvic lymph node involvement. Furthermore, the Systemic Immune-Inflammation Index (SII) appears to be a valuable marker to support decision-making in appropriate patient selection.
